# Endobronchial electrocautery wire snare prior to right middle sleeve lobectomy for adenoid cystic carcinoma of the lung: a case report

**DOI:** 10.1186/s44215-024-00149-3

**Published:** 2024-04-29

**Authors:** Hidenori Kawasaki, Hironobu Hoshino, Shoko Nakasone, Hiroki Kawabata, Tomofumi Yohena, Eriko Atsumi

**Affiliations:** 1 Department of Surgery, NHO Okinawa Hospital, 3-20-14 Ganeko, Ginowan City, Okinawa 901-2214 Japan; 2 Department of Pathology, NHO Okinawa Hospital, 3-20-14 Ganeko, Ginowan City, Okinawa 901-2214 Japan

**Keywords:** Adenoid cystic carcinoma, Endobronchial electrocautery wire snare, Sleeve lobectomy

## Abstract

**Background:**

Adenoid cystic carcinoma of the lung grows gradually, and spreads along the bronchial wall, often requiring tracheobronchoplastic procedure during surgery; however, incomplete resection occasionally occurs due to positive surgical margins. To avoid incomplete resection, effort should be exerted to confirm the extent of airway invasion of the tumor before surgery. Herein, we present the utility of combined treatment with bronchoscopic electrocautery wire snare for the endobronchial tumor prior to sleeve lobectomy with curative resection for patients with adenoid cystic carcinoma of the lung.

**Case presentation:**

A 56-year-old woman experienced a persistent cough 6 months prior. On an annual medical checkup, an abnormal lung shadow was noted. Chest computed tomography (CT) scan demonstrated right middle lobe atelectasis, and a round tumor shadow at the orifice of the right middle lobe bronchus, which protruded into the right intermediate bronchus, was observed. On bronchoscopy, a pedunculated endobronchial tumor in the intermediate bronchus was shown, and the middle lobe bronchus was completely obstructed. Initially, tumor resection via bronchoscopy was performed using an electrocautery wire snare under general anesthesia, and the tumor was pathologically diagnosed as adenoid cystic carcinoma of cT1aN0M0 stage IA. After tumor resection, the extent of tumor progression in the airway was assessed; subsequently, the patient underwent elective right middle sleeve lobectomy and lymphadenectomy. She survived without recurrence 7 years after surgery.

**Conclusion:**

We present a useful combined treatment strategy of bronchoscopic electrocautery wire snare prior to sleeve lobectomy for patients with endobronchial adenoid cystic carcinoma of the lung.

## Background

Adenoid cystic carcinoma of the lung is a relatively rare malignant tumor that arises from the bronchial glands. It grows gradually, and spreads along the bronchial wall, often requiring tracheobronchoplastic procedure during surgery; however, incomplete resection occasionally occurs due to positive surgical margins [1, 2]. Herein, we present the utility of combined treatment with bronchoscopic electrocautery wire snare for the endobronchial tumor prior to sleeve lobectomy with curative resection for patients with adenoid cystic carcinoma of the lung with obstructive atelectasis of the right middle lobe.

## Case presentation

**Fig. 1:**
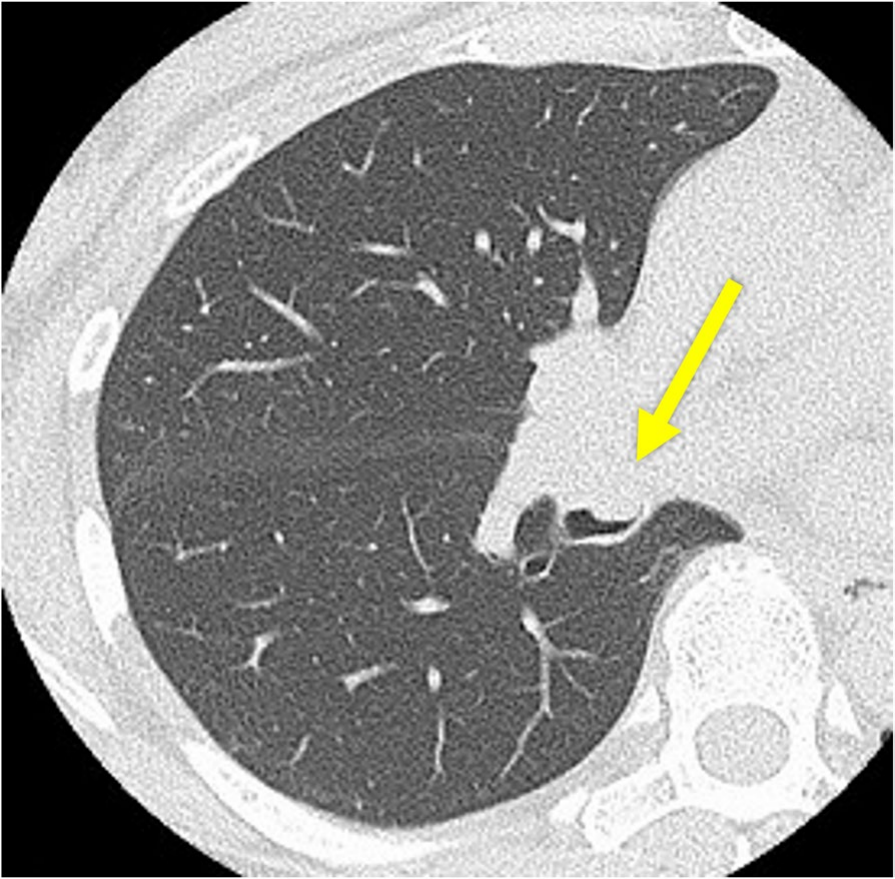
Chest computed tomography scan revealing atelectasis of the right middle lobe, and a round tumor present at the orifice of the right middle lobe bronchus that protruded into the right intermediate bronchus (arrow)

**Fig. 2:**
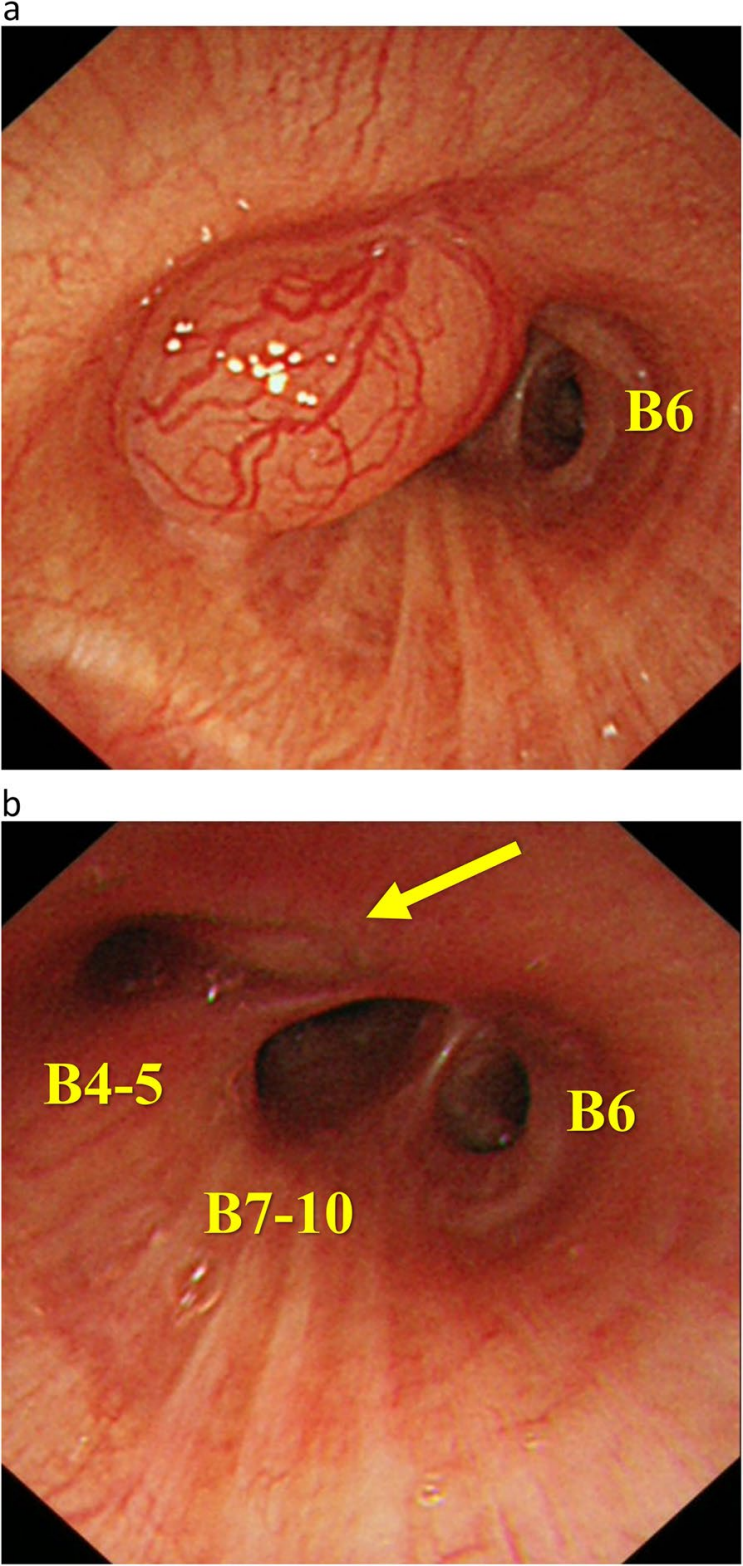
**a** Bronchoscopy depicts a smooth, mildly hypervascular endobronchial tumor, obstructing the middle lobe bronchus. **b** Bronchoscopy two weeks after endobronchial resection of the tumor shows the open middle lobe bronchus, and residual tumor in the middle lobe bronchial orifice (arrow)

**Fig. 3:**
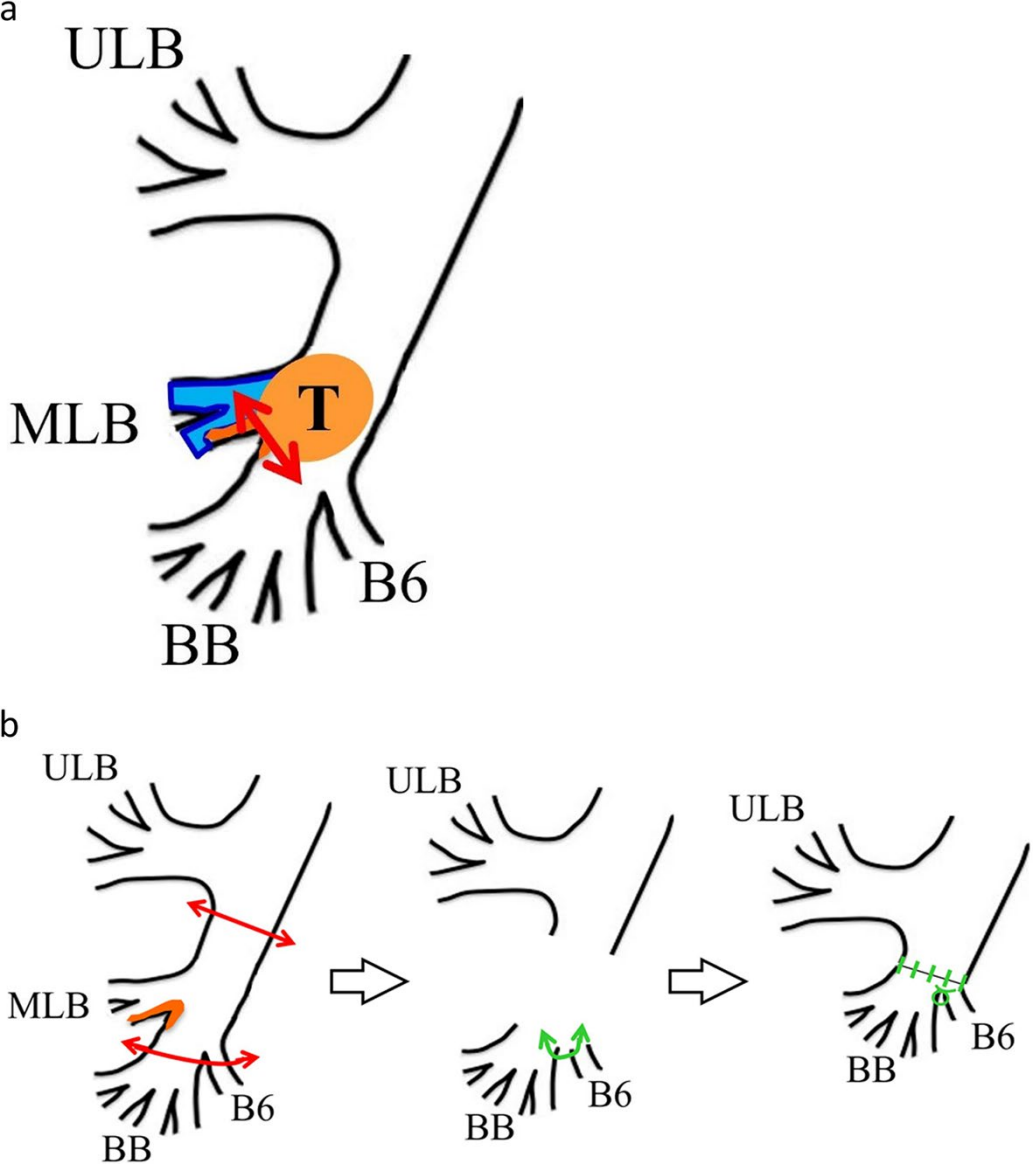
Schematic representation of the treatment procedure. **a** Endobronchial electrocautery wire snare resection of the endobronchial tumor. **b** Right middle sleeve lobectomy with a double-barrel-shaped maneuver. Abbreviations; ULB (right upper lobe bronchus), MLB (right middle lobe bronchus), BB (basal bronchus), T (endobronchial tumor)

The patient is a 56-year-old woman who never smoked. Her pertinent medical history includes hyperthyroidism and is on medication. She had a persistent cough for 6 months. She was diagnosed with pneumonia at a private clinic, and antibiotics were prescribed for symptom relief. Six months after the diagnosis of pneumonia, an increasing shadow density in the right lung hilum was noted on chest radiography during an annual medical checkup; thereafter, she visited a general hospital. In the general hospital, she was found to have right middle lobe atelectasis, and she was referred to our hospital for further examination. Chest computed tomography (CT) scan showed atelectasis of the right middle lobe and a round tumor shadow at the orifice of the right middle lobe bronchus that protruded into the right intermediate bronchus (Fig. 1). Additionally, two small pure ground glass nodule (GGN) shadows were found in the right upper (5 mm in size) and lower lobes (4 mm in size). Bronchoscopy demonstrated a pedunculated endobronchial tumor in the intermediate bronchus, and the middle lobe bronchus was completely obstructed. The tumor surface was smooth with mild hypervascularity (Fig. 2a). These findings also suggested benign or low-grade malignant bronchial tumor; therefore, performing an endoscopic resection was decided first for a definitive pathological diagnosis. Under general anesthesia, bronchoscopic tumor resection was conducted using an electrocautery wire snare. The wire loop was passed over the endobronchial tumor in the middle lobe bronchus. A blended electrocautery current was employed, and the obstructing tumor was successfully resected with minimal bleeding (Fig. 3a). The resected endobronchial tumor (14 mm in size) was confirmed as adenoid cystic carcinoma via histopathology. Two weeks after tumor resection, bronchoscopy showed open middle lobe bronchus, and residual tumor in the middle lobe bronchus orifice; thus, we decided to perform additional tumor resection (Fig. 2b). Further evaluation of distant metastases was performed. Brain magnetic resonance imaging showed no brain metastasis. Positron emission tomography/CT showed no fluorodeoxyglucose uptake in the orifice of the right middle lobe bronchus, including the GGN shadows in the right upper/lower lobe, which also showed the absence of metastases. Thus, the patient was diagnosed as clinical T1aN0M0 stage IA. Preoperatively, a right middle sleeve lobectomy was planned. Furthermore, partial resection was planned for the GGN shadows in the right upper lobe and lower lobe.

Surgical procedure (Fig. 3b). Under general anesthesia with double-lumen tracheal intubation, the patient was placed in the left lateral decubitus position, and posterolateral thoracotomy was started. The middle lobe pulmonary vessels and intermediate bronchus to lower lobe bronchus were dissected, and the middle lobe vessels were ligated and divided. The middle trunk bronchus and lower lobe bronchus were dissected and skeletonized. Initially, the intermediate bronchus was divided, followed by the division of the lower lobe bronchus, and the right middle lobe was removed. Each bronchial stump was then assessed for malignancy through rapid pathological examination and confirmed negative surgical margin. The lower lobe bronchus was divided more peripherally to the lower bronchi, and the bifurcation of the B6 bronchus (B6) and the basal bronchus (BB) was resected. Thus, B6 and BB were anastomosed in a double-barrel-shape maneuver, and an end-to-end anastomosis was performed to the middle trunk bronchus using 4–0 PDSII. Both the lateral edge of the B6 and BB anastomosis was anastomosed to the middle trunk bronchus using horizontal mattress sutures. An anastomotic line was reinforced with a pedicled intercostal muscle flap. Additionally, partial resection of the GGN shadows in the right upper and lower lobe was performed.

**Fig. 4:**
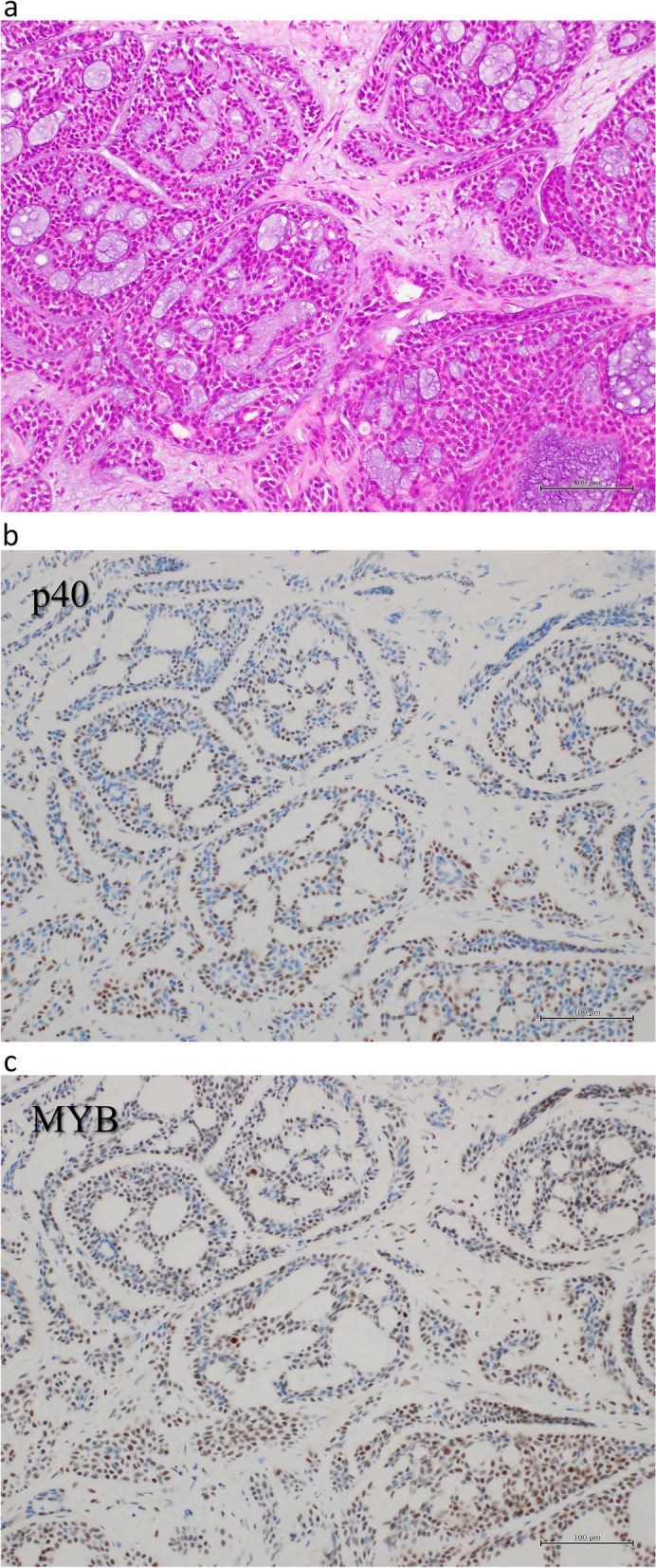
**a** Pathologically, the tumor revealed a cribriform and tubular pattern with nest of tumor cells. Multiple pseudocysts containing lightly basophilic matrix with some ductal differentiation were observed. **b**, **c** Immunohistochemistry, tumor cells were positive for p40 and MYB

Resected specimen revealed a residual tumor within the bronchial wall of the middle lobe bronchial orifice, measuring 13 mm along the bronchial wall. Pathologically, the tumor revealed a cribriform and tubular pattern with a nest of tumor cells. Multiple pseudocysts containing lightly basophilic matrix with some ductal differentiation were observed (Fig. 4a). Immunohistochemistry, tumor cells were positive for p40 and MYB (Fig. 4bc). The pathological diagnosis was adenoid cystic carcinoma, pT1N0M0 stage IA, with negative surgical margins. Partially resected GGNs in the right upper lobe (5 mm in diameter) and lower lobe (4 mm in diameter) were diagnosed as adenocarcinoma in situ and minimally invasive adenocarcinoma, respectively.

**Fig. 5:**
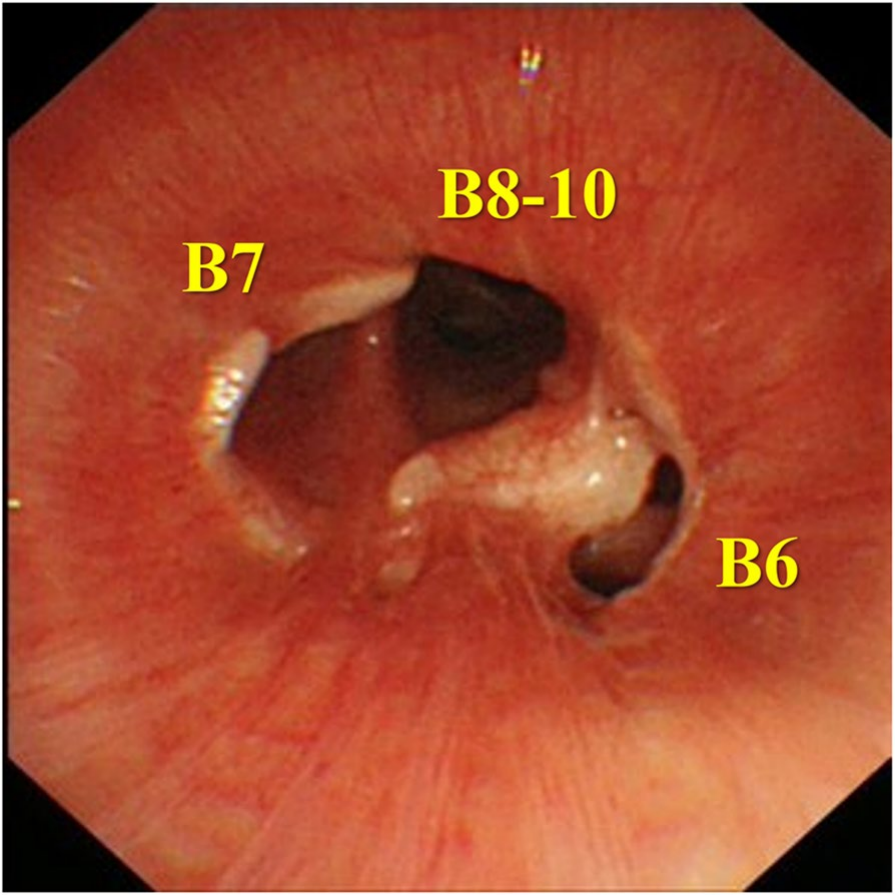
Postoperative bronchoscopy demonstrated satisfactory healing and patency

The patient’s postoperative course was uneventful without any complications, and she was discharged on the 9th postoperative day. Postoperative bronchoscopy 5 months later, revealed satisfactory healing and patency (Fig. 5). She survived without recurrence 7 years after surgery.

## Discussion

An adenoid cystic carcinoma often arises in the central airways. This tumor showed a gradual growth; however, it spreads along the bronchial wall and requires tracheobronchoplastic procedures in some surgical series. Intraoperative assessment of the extent of progression through gross findings and rapid pathological evaluation are required during surgery. However, in some cases, positive surgical margins are encountered from the wide bronchial wall invasion, resulting in incomplete resection [1, 2]. To avoid incomplete resection, effort should be exerted to confirm the extent of airway invasion of the tumor before surgery. Chest CT is mainly used to evaluate the extent of local progression of lung cancer prior to surgical resection. However, a detailed CT evaluation is often difficult for central airway-occurring tumors including adenoid cystic carcinoma. Bronchoscopy is essential to evaluate the progression of these tumors. However, evaluation of the peripheral side of the tumor remained difficult due to tumor obstruction, which makes it difficult to think of an appropriate treatment strategy. Removal of the airway-obstructing tumor to evaluate airway invasion at the periphery requires various available bronchoscopic interventions such as laser or electrocautery using a flexible or rigid bronchoscope [3]. The efficacy and safety of the Nd-YAG lasers and electrocautery have been reported for respiratory tract tumor treatment. The advantage of electrocautery wire snare, compared with Nd-YAG laser therapy, is faster tumor removal and its cost-efficacy. Wahidi et al. reported the utility of electrocautery and elucidated the treatment of 117 cases using endobronchial electrocautery wire snare, with a reported endoscopic improvement rate of 94%, symptom relief of 71%, radiological improvement of 78%, and severe complications of 0.8% [4].

Few literatures have been published on the efficacy and safety of lung resection with bronchoplasty after airway intervention [5–7]. Studying the effects of airway interventions on the tracheal wall, van Boxem et al. evaluated the degree of damage and bronchial wall healing after photodynamic, Nd-YAG laser and electrocautery therapy; electrocautery produced limited bronchial mucosa damage, few minimal scarring, and subepithelial fibrosis [8]. These results indicate that even if bronchoplasty is performed after electrocautery wire snare, there is minimal damage to the anastomosis. Pandey et al. describe a case of bronchial carcinoid treated via bronchoscopic snaring of polypoid bronchial carcinoid followed by wedge bronchoplastic lobectomy [5]. Kawasaki et al. reported improvement in atelectasis and confirmation of the extent of infiltration in the bronchus after a left bronchial tumor resection with left atelectasis using a snare, followed by left lower lobectomy with wedge bronchoplasty [7]. In this report, we present bronchoscopic electrocautery wire snare resection of the endobronchial tumor prior to middle sleeve lobectomy for patients with adenoid cystic carcinoma of the lung with obstructive atelectasis of the right middle lobe. This strategy has the following advantages. A large sample can be extracted, and an accurate pathological diagnosis is more reliable. Moreover, it relieves airway obstruction and provides a more rapid symptom improvement. After tumor resection, the extent of tumor progression in the airway can be assessed, and an optimal treatment can be selected. Furthermore, additional bronchoplastic surgery can be safely performed after radiofrequency resection of the bronchial tumor.

## Data Availability

The authors confirm that the findings of this case are available within the published article.

## Abbreviations

CT: Computed tomography
GGN: Ground glass nodule
BB: Basal bronchus
